# Involvement of CD11b integrin in the alteration of metabolic factors after phorbol ester stimulation of human myeloid leukemia cells

**DOI:** 10.1186/1478-811X-10-13

**Published:** 2012-07-11

**Authors:** Katharina Mandel, Anna Otte, Ralf Hass

**Affiliations:** 1 Biochemistry and Tumor Biology Lab, Gynecology Research Unit, Department of Obstetrics and Gynecology, Medical University, Hannover, Germany; 2 Biochemistry and Tumor Biology Lab, Gynecology Research Unit, Department of Obstetrics and Gynecology (OE 6410), Medical University Hannover, Carl-Neuberg-Str. 1, 30625, Hannover, Germany

**Keywords:** U937 leukemia cells, Differentiation, Adherence, Telomerase, Proteases, Signalling

## Abstract

Previous work has demonstrated that phorbol ester (TPA)-induced adherence of human U937 myeloid leukemia cells can be blocked upon down-modulation of the β2-integrin CD11b after stable transfection of U937 cells with a pMTH1 vector-containing the CD11b gene in antisense orientation (asCD11b-U937) *[Otte* et al.*, (2011)]*. In the present study, alterations in metabolism-associated factors, particularly intra- and extracellular proteases were investigated. A measurement of telomerase activity in the leukemic cells revealed continuously decreasing telomere adducts within 72 h of TPA treatment in pMTH1-U937 cells. In contrast, telomerase activity sustained in asCD11b-U937 upon TPA-induced differentiation. Flow cytometric analysis confirmed unchanged CD11b levels in TPA-induced asCD11b-U937 in contrast to elevated levels in pMTH1-U937 whereby the expression of other β2-integrins including CD11a, CD11c and CD18 was increased in both populations after TPA treatment. Moreover, adherent pMTH1-U937 demonstrated the expression of monocytic differentiation markers including F4-80 and CD14 and an increased MIP-1α production which remained at low or undetectable in TPA-induced asCD11b-U937. These effects indicated an altered response of the different cell populations to the TPA-induced differentiation process. Indeed, Western blot analysis revealed differences in the expression levels of intracellular metabolic factors including MnSOD and p97/VCP and after measurement of 20 S proteasomal proteolytic activity. In addition, increased levels of extracellular metabolic factors including the matrix metalloproteinases MMP-1, MMP-7 and MMP-9 were observed in pMTH1-U937 cells in contrast to unaltered levels in asCD11b-U937 cells.

## Introduction

The human U937 myeloid leukemia cell line represents an *in vitro* model for monocyte/macrophage-like differentiation and retrodifferentiation [1-5]. A variety of molecular effects by differentiation-inducing agents such as the phorbol ester derivate 12-O-tetradecanoyl-phorbol-13-acetate (TPA) on cell adherence and filament expression in U937 cells have been extensively characterized [6,7]. Thus, the non-adherent growing and like tumor cells autonomously proliferating U937 wild-type population can be stimulated by TPA to differentiate along the monocyte/macrophage pathway which is associated with induced adherence and cessation of cell growth. The TPA-mediated attachment of differentiating U937 cells is accompanied by an enhanced expression of the β2-integrins CD11a, CD11c, CD18, and particularly CD11b [6]. These integrin compounds are cell membrane-associated glycoproteins whereby CD11a, CD11b and CD11c represent separate α-subunits to associate with the common β-subunit CD18, respectively. The appropriate heterodimeric protein complex forms a functional β2-integrin which is involved in the formation of cell-to-cell contacts and intercellular communication processes [8]. Moreover, junctional adhesion molecules including ICAMs can associate through their extracellular domains with functional β2-integrins on adjacent cells, contributing for example to the regulation of leukocyte-endothelial cell interactions [9].

Previous work has demonstrated that a differentiation-defective subclone of the U937 cell line, termed TUR (TPA-U937-resistant), fails to express significant levels of CD11b after TPA treatment [10]. Concomitantly, these human TUR leukemia cells are unable to attach and continue to proliferate in response to a phorbol ester stimulation [11] indicating that CD11b displays a differentiation-associated function beyond an involvement in the regulation of cell attachment. Indeed, previous work has demonstrated that a down-modulation of the CD11b integrin fails to develop certain markers of a monocytic phenotype following exposure to the differentiation-inducing TPA [1,12]. Such a differentiation program along the monocyte/macrophage-like lineage in TPA-treated U937 cells requires significant metabolic changes and other studies have shown that this process is accompanied by alterations in the expression and activity of metabolizing factors including the 20 S proteasome [13], manganese peroxidase [14] and the valosin-containing protein VCP/p97 [7]. Moreover, the acquired adherence of myelocytic cells during monocytic maturation is paralleld by a restructure of the extracellular matrix involving a variety of matrix metalloproteinases such as MMP-1, MMP-7 and MMP-9 [14].

Whereas previous work has determined the role of CD11b integrin-mediated cell attachment and cell cycle progression within a monocytic differentiation program [1], little is known about intracellular and extracellular metabolic enzymes which may affect restructure of the extracellular matrix and are also relevant for maturation along the monocytic lineage.

It was therefore of interest in the present study, to examine the role of down-modulated CD11b integrin on metabolic factors represented by the expression levels and activation of distinct intracellular and extracellular protease systems after phorbol ester treatment.

## Material and methods

### Cell culture

Human U937 myeloid leukemia cells (American Type Culture Collection #CRL-1593.2) were cultured in RPMI 1640 containing 10% of heat-inactivated fetal bovine serum, 100 units/ml penicillin, 100 μg/ml streptomycin, and 2 mM L-glutamine in a 37°C humidified atmosphere with 5% CO_2_. Moreover, U937 cells stably transfected with the pMTH1 vector (pMTH1-U937) and U937 cells stably transfected with the pMTH1 vector containing the CD11b gene in antisense orientation (asCD11b-U937) [12] were cultured under similar conditions. The different cell populations were incubated with 5nM of the differentiation-inducing agent 12-O-tetradecanoylphorbol-13-acetate (TPA) (Sigma Chemie GmbH, Taufkirchen, Germany) for different time points as indicated.

### Telomerase assay

Using a radioactive assay the activity of this nuclear enzyme in U937 cells, pMTH1-U937 and asCD11b-U937 cells was detected by TRAPeze telomerase detection kit (Millipore, Beverly, MA, USA). Briefly, homogenates of pMTH1-U937 and asCD11b-U937 cells after TPA exposure at distinct time points was resuspended in CHAPS lysis buffer and combined with a reaction mixture including a [γ-32P] ATP radiolabeled TS primer previously labelled by T4-polynucleotide kinase (NEB, Beverly, MA, USA). Evaluation and adjustment of equal protein was performed using the Bradford method (Bio-Rad Inc., Richmond, CA, USA). The different pMTH1-U937 and asCD11b-U937 protein samples were subjected to PCR amplification using Taq DNA polymerase (NEB, Beverly, MA, USA) according to the manufacturer’s instructions. Following the addition of loading dye the amplified DNA samples were separated in a 10% non-denaturing polyacrylamide gel. Thereafter, the gel was dried and the radiolabelled telomere bands were visualized in a PhosphoImager (Storm 820, Amersham Biosciences).

### Flow cytometry analysis

Steady state cultures of pMTH1-U937 control cells and asCD11b-U937 were harvested, washed in phosphate-buffered saline/bovine serum albumin (PBS-BSA), and plated at 1x10^6^ cells/well in round-bottom microtiter plates (BD Biosciences GmbH, Heidelberg, Germany). Fc-receptors were blocked by addition of 20 μl of human IgG, diluted to 10 mg/ml for 30 min at 4°C. The cells were washed with PBS-BSA and aliquots were incubated with a 1:50 dilution of the fluorescein isothiocyanate (FITC)-conjugated monoclonal antibodies anti-human CD11a (Serotec Ltd., Oxford, U.K.) and anti-human CD18 (Serotec) for 1 h/4°C in the dark. Furthermore, aliquots of the cells were also incubated with a 1:50 dilution of a RPE-labeled monoclonal anti-human CD11b (Biozol GmbH, Eching, Germany), anti-human CD11c (Serotec), anti-human CD14 (Serotec), and the rat anti-mouse F4-80 antibody (Boehringer Mannheim) for 1 h/4°C, respectively. After three washes with PBS/BSA the cells containing the non-labelled antibodies were incubated with a secondary FITC-conjugated rabbit anti-mouse (Dianova GmbH, Hamburg, Germany), and in case of the F4-80 with a FITC-conjugated sheep anti-rat (Dianova) antibody for 1 h/4°C, respectively. Following antibody incubation all samples were washed twice with PBS-BSA and flow cytometry was performed in a Galaxy FACSan (Partec) using FloMax analysis software (Partec).

### Measurement of the nuclear proteasomal proteolytic activity

About 5 × 10^5^ U937 cells, pMTH1-U937 control cells and asCD11b-U937 cells were exposed to 5nM TPA for 4 h, 8 h, 24 h, 48 h and 72 h, respectively. At the time points indicated the different cell populations were harvested and enrichment of nuclei was performed as described previously [7] to minimize contamination of other cellular components. The isolated nuclei were lysed by 5 freeze-thawing-cycles in 10 mM HEPES (pH 7.5), 24 mM KCl, 10 mM MgCl_2_ and 0.5 mM DTT. The proteasome activity in nuclear lysates was measured as described previously [15]. Briefly, the peptidase activity toward the hydrophobic fluorogenic peptide succinyl-leucine-leucine-valine-tyrosine-methylcoumaryl-amide (suc-LLVY-MCA) was measured by incubation with 50 mM Tris–HCl (pH 7.8), 20 mM KCl, 5 mM MgOAc, 0.5 mM DTT and 200 μM suc-LLVY-MCA for 1 h at 37°C in the presence and absence of 10 μM lactacystin. After incubation for 2 h at 37°C, the reaction was stopped by the addition of an equal volume of ice-cold ethanol and a 10-fold volume of 125 mM sodium borate (pH 9.0). Thereafter, fluorescence intensity was determined at 380 nm excitation and 440 nm emission wavelength.

### Measurement of Mip-1α production

About 5 × 10^5^ U937 cells, pMTH1-U937 control cells and asCD11b-U937 cells were exposure to 5nM TPA for 4 h, 8 h, 24 h, 48 h and 72 h, respectively. Following incubation, 100 μl aliquots of the appropriate supernatant were transferred into an anti-Mip-1α-precoated microtiter plate and analysed for Mip-1α by enzyme-linked immunosorbent assay (ELISA) according to the manufacturer’s instructions (Tebu-Bio, Offenbach, Germany). After the stop solution was applied to each sample, absorption at 450 nm was immediately measured in a Multiskan EX ELISA reader (ThermoFischer Scientific, Langenselbold, Germany).

### Immunoblot analysis

The immunoblot analysis was performed in accordance to a closely-related recent study [1]. Thus, untreated and 5nM TPA-stimulated pMTH1-U937 and asCD11b-U937 cells were washed three times in ice-cold PBS and lysed in a buffer containing 10 mM Tris–HCl (pH 7.6), 140 mM NaCl, 10 mM EDTA, 1% (v/v) NP-40 with the addition of 10 μg/ml aprotinin, 10 μg/ml leupeptin, and 1 mM phenylmethylsulfonylfluoride (PMSF) (all from Sigma). Protein concentration was adjusted using the colorimetric BCA-assay (Perbio Science Deutschland, Bonn, Germany), subjected to SDS-polyacrylamide gel electrophoresis and transferred to a PVDF membrane (Millipore GmbH, Schwalbach, Germany). The membranes were blocked with PBS containing 5% FCS and 0.05% Tween-20 (PBS/Tween). After washing four times with PBS/Tween, the membranes were incubated with the primary antibodies and subsequently stripped. Following primary antibody incubation (polyclonal anti-MnSOD (Upstate Inc., Lake Placid, NY, USA); polyclonal anti-p97/VCP (Novus Biologicals Inc., Littleton, CO, USA); monoclonal anti-proteasome, clone MCP231 (Enzo Life SciencesGmbH, Lörrach, Germany); polyclonal anti-MMP-1 (Biomol); monoclonal anti-MMP-7, clone 111433 (Biomol); polyclonal anti-MMP-9 (Biomol) and monoclonal anti-β-actin, clone AC-15 (Sigma) for 2 h/37°C, the membranes were washed four times with PBS/Tween and incubated with the appropriate horseraddish peroxidase-conjugated secondary antibody (all from Santa Cruz Biotechnology, Santa Cruz, CA) for 1 h/37°C. The membranes were washed with PBS/Tween and visualized by autoradiography using the ECL-detection kit (GE Healthcare, München, Germany).

### Gelatin zymography for detection of MMP gelatinase activity

Following exposure of 10^7^ U937 cells, pMTH1-U937 cells and asCD11b-U937 cells to 5nM TPA for 72 h, respectively, the cells were washed in PBS and incubated in 20 ml RPMI-1640 medium containing only 0.1% FCS for 24 h. Similarly, non-induced controls were seeded in the same media and cell density for 24 h. Thereafter, the conditioned media supernatants were collected and concentrated 18-fold using Amicon Ultra-4 Centrifugal Filter Devices (Millipore, Carrigtwohill, Ireland) according to the manufacturer’s instructions. These concentrated media samples were used in a zymographic assay to detect any gelatinase proteolytic activity corresponding to certain MMPs. Thus, 25 μl aliquotes of the samples were mixed 2:1 (v/v) with non-reducing sample buffer (10 mM Tris (pH 6·8), 1% SDS, 10% glycerol and 0.02% bromophenol blue) and subjected to SDS-PAGE containing 2 mg/ml of gelatine (Sigma, Steinheim, Germany). Electrophoresis was performed for 30 min at 60 V followed by 120 min at 125 V. The gels were washed twice in 2.5% Triton X-100 on a vertical shaker and five times with H_2_O. Thereafter, the gels were incubated with fresh MMP enzyme buffer (50 mM Tris–HCl, pH 7, 5 mM CaCl_2_) overnight at 37°C. Finally, the gels were stained with 0.4% Coomassie blue (methanol/acetic acid/H_2_O (40:10:50)) for 1 h and destained with 10% acetic acid in 50% methanol until bands were visualized. The proteolytic activity was detected by the appearance of light bands against the dark blue background.

## Results

**Figure 1:**
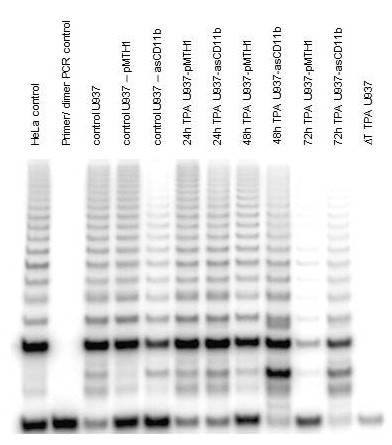
**a: Telomerase activity.** Telomerase activity was detected by TRAPeze telomerase detection kit (Millipore, Beverly, MA, USA). Thus, radiolabeled telomere adducts were measured in the wild-type U937 control cells as compared to pMTH1-U937 and asCD11b-U937 cells following culture in the absence and in the presence of 5nM TPA for 24 h up to 72 h, respectively. One representative telomerase assay out of three similar ones is presented. **b: Proliferation assay.** About 2 × 10^4^ cells (either U937, pMTH1-U937 or asCD11b-U937) were incubated in 96-well microtiter plates in the presence or absence of 5nM TPA for up to 72 h. The cells were radiolabelled with about 0.5 μCi/well [^3^ H] thymidine for 15 h and at the time points indicated, the cells were harvested and the radioactivity was quantified in a β-scintillation counter. The incorporated [^3^ H] thymidine was calculated as percentage of the appropriately untreated control cells at each time point which were set to 100%. Data represent the mean ± s.d. (n = 10).

The tumor cell properties of wild-type U937 control cells, pMTH1-U937 and asCD11b-U937 human myeloid leukemia cells was measured by the telomerase activity assay and after induction of differentiation with 5nM TPA (Figure 1a). Thus, pMTH1-U937 cells demonstrated a detectable constitutive telomerase activity whereby TPA treatment abolished this telomerase activity after 72 h (Figure 1a). In contrast, asCD11b-U937 transfectants exhibited a slightly decreased telomerase activity as compared to pMTH1-U937, however, this telomerase activity increased 24 h and 48 h after exposure to the tumor promoter TPA and prevailed at lower levels after a 72 h TPA incubation (Figure 1a). These findings indicated a sustained tumorigenic potential of asCD11b-U937 cells in the presence of the phorbol ester whereby TPA may also affect further known signalling pathways such as PKC activation [11].

The proliferation of wild-type U937 and pMTH1-U937 cells as measured by ^3^ H]thymidine incorporation revealed a significant reduction by about 75% to 85% after 72 h of TPA treatment (Figure 1b) which is in agreement with previous studies demonstrating TPA-mediated growth inhibition [1,10,11]. In contrast, phorbol ester exposure to asCD11b-U937 cells was associated with a sustained ^3^ H]thymidine incorporation in more than 80% of the cells (Figure 1b).

**Figure 2:**
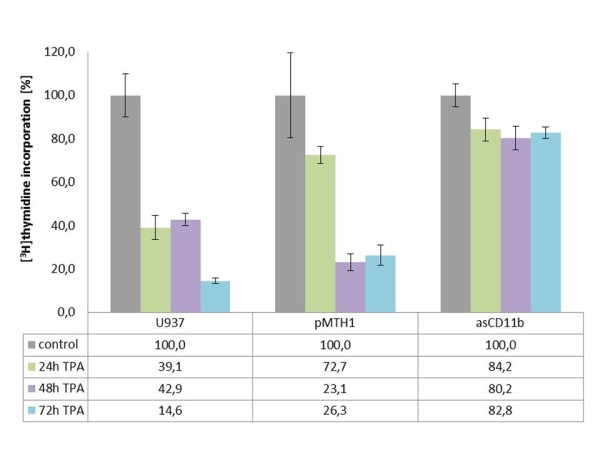
**Flow cytometry analysis of cell surface markers.a**) Wildtype U937 cells, pMTH1-U937 and asCD11b-U937 were compared with respect to the expression of the CD11b antigen in both, steady-state culture and following a 72 h exposure to 5nM TPA. An IgG1 subclass staining serves as a control which was not altered in the different populations. **b**) Untreated pMTH1-U937 (pMTH1) and untreated asCD11b-U937 (asCD11b) cells were cultured in either uncoated or 2% (w/v) agarose-precoated cell culture dishes. Following 72 h of incubation with 5nM TPA, respectively, the cells were harvested and flow cytometry analysis was performed in the different populations with the β2-integrin subunit antibodies CD11a, CD11c and CD18 as well as with the monocytic differentiation markers CD14 and F4-80. The relative fluorescence intensity represents the fold induction which has been normalized separately for the appropriate control cell population. Data represent the mean ± s.d. of three measurements.

In order to examine the consequences of CD11b down-modulation on some surface molecules during phorbol ester treatment, FACS analysis was performed to evaluate the integrin levels and expression of distinct monocytic markers in the pMTH1-U937 control vector and asCD11b populations (Figure 2). There was little if any detectable constitutive expression of CD11b in either wild-type U937, pMTH1-U937 or asCD11b-U937 cells (Figure 2a). However, phorbol ester treatment revealed an induction of CD11b antigen expression to 70.7% and 71.7% after 72 h of in U937 wild-type and pMTH1-U937 cells, respectively, whereas asCD11b-U937 demonstrated only an induction of 18.0% (n = 3) (Figure 2a). Other members of the β2-integrin family, including CD11a, CD11c and the β-subunit CD18 were expressed in an enhanced fashion after TPA treatment of pMTH1-U937 which is in concert with previous findings in TPA-treated U937 cells [5]. These levels were even further elevated in TPA-stimulated pMTH1-U937 cells cultured on 2% agarose which non-specifically blocks cell-to-substrate adherence (Figure 2b). Similar findings were observed for CD11a, CD11c, and CD18 in asCD11b-U937, although the induction levels of CD11a were more pronounced than in pMTH1-U937, whereas less induction was observed for CD11c and CD18 when compared to pMTH1-U937 (Figure 2b).

Previous work has demonstrated that phorbol ester treatment of U937 cells is also associated with enhanced expression of monocytic differentiation markers including CD14 [6]. Similar results were obtained in pMTH1-U937 cells. Incubation of these cells with TPA in the absence or presence of an agarose layer demonstrated a significant induction of monocytic epitopes recognized by anti-CD14 and anti-F4-80 antibodies, respectively (Figure 2b). In contrast, there was little if any induction of the monocytic surface markers detectable in TPA-treated asCD11b-U937 cells on either plastic or agarose (Figure 2b). Together, these data demonstrated a reduced induction of both, CD11b and certain monocytic differentiation markers in antisense CD11b transfectants following TPA stimulation suggesting differentiation defects and functional alterations due to the down-modulation of the CD11b integrin. This hypothesis is supported by the measurement of the macrophage inflammatory protein-1-alpha (Mip-1α) release as a functional marker of activated monocytic/macrophages (Table 1). Whereas wild-type U937 and pMTH1-U937 cells progressively increased the Mip-1α production to about 372 ng/ml and 344 ng/ml within 72 h of TPA treatment, respectively, the amount of Mip-1α remained below detection limit in asCD11b-U937 cells and reached about 1 ng/ml only after 72 h (Table 1).

**Table 1:** Measurement of Mip-1α release

Production and secretion of Mip-1α [ng/ml] following stimulation with 5nm TPA						
	0h	4h	8h	24h	48h	72h
U937	n.d.	0.21 ± 0.16	19.75 ± 0.55	302.06 ± 22.00	331.11 ± 15.26	372.47 ± 35.47
pMTH1-U937	n.d.	0.21 ± 0.16	23.53 ± 1.66	340.45 ± 24.41	334.87 ± 7.89	344 ± 33.30
asCD11b-U937	n.d.	1.20 ± 0.11	3.06 ± 0.03	3.20 ± 0.06	2.10 ± 0.08	1.06 ± 0.15

About 5x10^5^ U937 cells, pMTH1-U937 and asCD11b-U937 cells were incubated in the absence or presence of 5nM TPA for up to 72 h, respectively. At the time points indicated aliquots from the cell supernatants were applied to a Mip-1α ELISA. The results are expressed as ng Mip-1α/ml with a detection limit below 6 pg/ml (n.d. = not detectable (below detection limit)). Data represent the mean ± s.d. of one experiment performed in triplicate.

**Figure 3:**
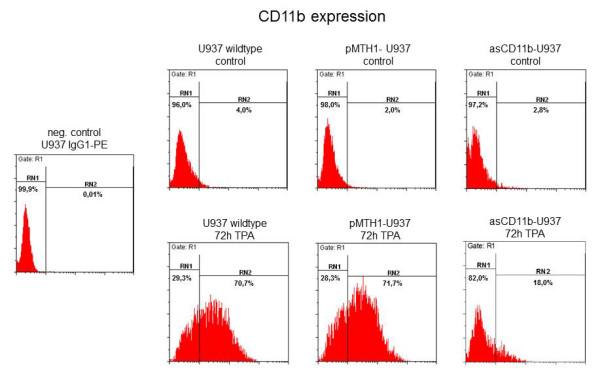
**Western blot analysis.** The pMTH1-U937 (pMTH1) and asCD11b-U937 (asCD11b) cells were cultured in the absence and in the presence of 5nM TPA for 24 h up to 72 h, respectively. Thereafter, the different populations were harvested, lyzed and 40 μg of total cellular protein was separated by 10% SDS-PAGE followed by Western blotting. **a**) For the expression of the metabolic enzymes MnSOD, p97/VCP and the 20 S proteasome (α-subunits), gels were subsequently stripped corresponding to a β-actin loading control. **b**) Analysis of the expression patterns of different extracellular matrix proteolytic enzymes were tested using antibodies against MMP-1, MMP-7 and MMP-9 for matrix restructuring. The unaltered expression level of β-actin serves as a loading control.

In order to further test the hypothesis of differentiation defects and functional alterations due to the down-modulation of the CD11b integrin, Western blots were applied to analyze expression levels of intra- and extracellular protease systems as potential metabolic factors. Whereas phorbol ester treatment and a maturation along the monocyte/macrophage lineage is associated with enhanced metabolic activities in distinct subcellular compartments, expression of mitochondria-associated manganese superoxide dismutase (MnSOD) which catalyzes the dismutation of superoxide into oxygen and hydrogen peroxide, revealed enhanced protein levels in pMTH1-U937 cells as compared to asCD11b-U937 cells (Figure 3a). Similarly, expression of the AAA ATPase valosin-containing protein (VCP/p97) which contributes to protein degradation and cell cycle regulation was elevated in pMTH1-U937 cells following TPA exposure (Figure 3a). Alpha-subunits of the 20 S proteasome detected by the appropriate antibody demonstrated a nearly unaltered expression in the different cultures of pMTH1-U937 and asCD11b-U937 cells, respectively (Figure 3a). Extracellular matrix proteinases were also tested since previous work has implicated an involvement of these factors in the cell attachment of human myeloid leukemia cells during the course of phorbol ester-induced monocytic differentiation [14]. Thus, the matrix metalloproteinases MMP-1, MMP-7 and MMP-9 were expressed in an enhanced fashion in TPA-treated pMTH1-U937 in contrast to asCD11b-U937 cells with little if any detectable MMP induction (Figure 3b).

**Figure 4:**
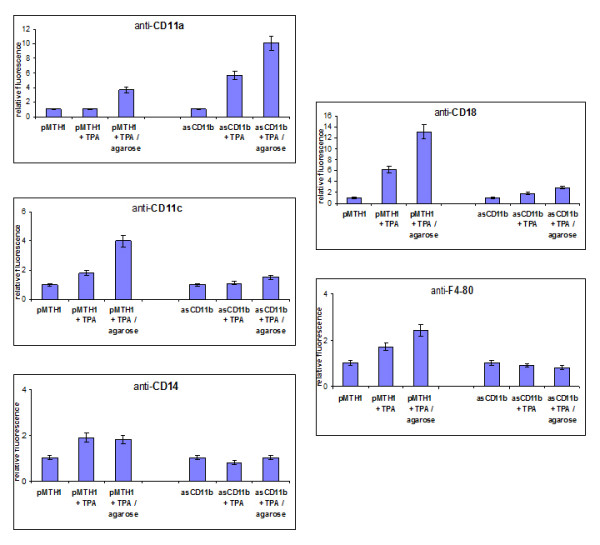
**20S proteasomal proteolytic activity.** The pMTH1-U937 and asCD11b-U937 cells were treated with 5nM TPA for 4 h up to 72 h. At the time points indicated the cells were harvested and nuclear extracts were measured by a fluorometric proteolytic assay to obtain the 20 S proteasomal activity. The relative proteasomal activity of pMTH1-U937 control cells in steady-state was set to 100%. Data represent the mean ± s.d. of three independent experiments.

While previous work has demonstrated that unaltered amounts of 20 S proteasome proteins may still represent different activity levels due to altered proteasomal regulations [16], the metabolic activities were measured for the 20 S proteasome (Figure 4). Indeed, the steady-state level (control) of pMTH1-U937 exhibited a nearly 2-fold enhanced proteasomal activity as compared to the asCD11b-U937 cells (Figure 4). Following TPA treatment, pMTH1-U937 cells revealed a progressively reduced 20 S proteasomal activity within 72 h in contrast to the asCD11b-U937 cells which demonstrated a significant increase in the proteasomal proteolytic activity (Figure 4). While the results of pMTH1-U937 cells are in agreement with those of TPA-induced wild-type U937 cells [13], these findings suggested that down-modulation of the CD11b integrin significantly altered the proliferative and differentiation capacity of the cells associated with marked intra- and extracellular metabolic changes.

**Figure 5:**
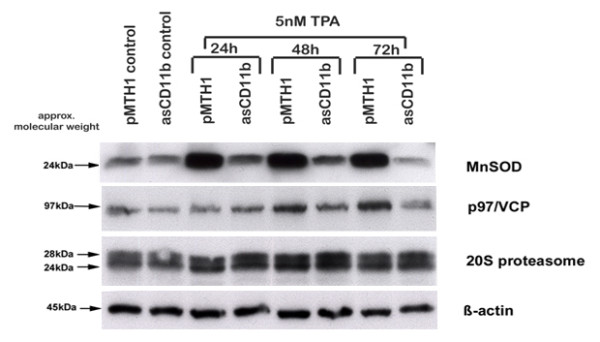
**Zymographic assay of gelatinase activity.** Medium supernatants of 20 ml culture medium (medium control) as well as 20 ml conditioned medium from 10^7^ U937 cells, pMTH1-U937 cells and asCD11b-U937 cells in the absence or presence of 5nM TPA for 72 h, respectively, were 18-fold concentrated and subjected to SDS-PAGE containing 2 mg/ml of gelatine. Following incubation with MMP enzyme buffer, the gels were stained with 0.4% Coomassie blue and afterwards destained again to visualize the appearance of gelatinase activity by light bands against the dark background. The molecular weight markers on both sides of the gels indicate the size of the MMPs exhibiting gelatinase activities.

Metabolic activities were also tested for the gelatinase activity of MMPs by performing a zymographic assay (Figure 5). Whereas U937 controls, pMTH1-U937 and asCD11b-U937 did not exhibit significant gelatinase activity as compared to the medium control, a 72 h TPA-treatment was associated with a significantly detectable gelatinase activity in U937 and pMTH1 cells which corresponded to the approximately 92 kDa MMP-9 and further proteases of about 43 kDa and 45 kDa according to the molecular weight standard on both sides of the gel (Figure 5). In contrast, gelatinase activities in asCD11b-U937 cells remained at undetectable levels after phorbol ester exposure (Figure 5). These activity levels are in agreement with the TPA-induced MMP data in Figure 3b by correlating significantly enhanced gelatinase activity with the elevated MMP expression levels in TPA-treated pMTH1-U937 in contrast to asCD11b-U937.

## Discussion

Phorbol ester treatment of non-adherent human myeloid leukemia cells is associated with cell attachment to form 3-dimensional cell aggregates, growth arrest and monocytic differentiation, and conversely, the reversible process of retrodifferentiation and rejuvenation reveals the loss of previously acquired monocytic features, a regained proliferative capacity and cell detachment to form again a single cell suspension of monoblastoid precursor cells [4,5]. Whereas these findings suggested a tight regulatory relationship between adherence, cell growth and metabolic changes, previous work has documented a predominant role of the β2-integrin CD11b in TPA-induced adherence of human leukemic cells [6]. Moreover, this role was abolished after down-modulation of β2-integrin CD11b in antisense transfectants by sustained telomerase and proliferative activity and failed monocytic differentiation after TPA treatment.

A functional β2 integrin composition in monocytic cells is involved in adhesion, transendothelial migration and phagocytosis [17] and it can relay downstream signals for cell polarity via the small Rho GTPase Cdc42 in neutrophils [18], however, the detailed signalling mechanisms of the β2-integrins via downstream kinases to contribute to a regulatory balance between adhesion-mediated cell cycle progression and a paralleled differentiation program remain unclear.

Recent data suggested that during an inflammatory response the functional CD11b/CD18 integrin complex, which represents the complement receptor-3, mediates phagocytosis depending on the activation of certain Rho GTPases [19]. Moreover, an inflammation-accompanied activation of CD11b/CD18 in murine peripheral blood mononuclear cells relayed a downstream signalling cascade via FAK/PI3K/Akt/NF-kappaB with subsequent expression of pro-inflammatory cytokines [20]. Thus, enhanced cytokine release such as the increased macrophage inflammatory protein-1-alpha (Mip-1α) production in TPA-activated pMTH1 transfectants and U937 cells indicated a functional β2 integrin signalling cascade via downstream kinases and transcription factors and conversely, these effects appear to be abolished in asCD11b cells which were unable to produce significant amounts of Mip-1α.

The failure of asCD11b cells to respond to TPA-activated kinases and phosphatases with the induction of a monocytic differentiation program in contrast to pMTH1 transfectants and U937 cells may elevate the superoxide anion production and DNA damage products [21-23] due to the inability of macrophage-like maturation and appropriate metabolic responses. Indeed, these effects may accumulate oxidative stress products in asCD11b cells and increased protein damage since the expression of reactive oxygen species (ROS)-metabolizing enzymes such as MnSOD or the expression of the chaperone p97/VCP in ubiquitin-dependent proteasomal degradation remained at low levels in contrast to the pMTH1 transfectants, respectively. Previous work has demonstrated that CD11b stimulation in monocytes can promote IL8 and Mip-1α production via a proteasome-dependent NF-κB activation which may in part explain the low steady-state proteasomal activity levels in the asCD11b transfectants most probably lacking a sufficient threshold of CD11b stimulation [24]. Moreover, a significantly increasing proteasomal proteolytic activity was observed despite of unchanged protein expression levels of the 20 S proteasome α-subunits. Whereas the 20 S proteasome represents a large 700 kDa multicatalytic and multisubunit proteinase complex, these findings could indicate possible alterations in the β-subunit composition. Other findings have demonstrated that poly(ADP-ribose) polymerase-1 (PARP-1) displays regulatory effects on the 20 S proteasomal activity [14,16,25] and similar mechanisms may contribute to the effects observed in asCD11b-U937. Indeed, the 20 S proteasome data are in agreement with previous work, demonstrating an unchanged proteasome protein expression despite of a markedly elevated proteasomal proteolytic activity in ROS-induced U937 cells. These effects were due to an oxidative stress-induced altered proteasomal regulation [13,16] suggesting a continuous accumulation of damaging products in TPA-exposed asCD11b-U937 cells which eventually leads to massive apoptosis in a certain part of the population as documented earlier [1,12]. An accumulation of damage products, e.g. ROS, may also activate apoptosis-associated proteases (e.g. caspases) for PARP-1 cleavage [26]. In contrast, the progressive decline of proteasomal activity in TPA-treated pMTH1 transfectants is also substantiated by previous reports during phorbol ester-induced differentiation of U937 cells [13].

In concert with the regulation of these intracellular proteolytic systems, extracellular matrix metalloproteases such as MMP-1, MMP-7 and MMP-9 have been reported to play a pivotal role in human tumor systems and autoimmune disorders to provide and actively change an appropriate microenvironment for the tumor cells as well as relay intracellular signals by involving cytokine receptors such as CXCR4 [27-29]. Thus, previous work in TPA-induced differentiating myeloid leukemic cells has demonstrated a strong induction of these matrix metalloproteases [14] which is in accordance with differentiating pMTH1 U937 cells and in contrast to the asCD11b transfectants. These findings reflect a relevance for β2-integrin-mediated signalling with the extracellular matrix substantiating the importance of an appropriate tumor cell microenvironment and restructure [30-32]. Moreover, these effects appear to be specific for CD11b since alternative β2-integrins such as CD11a/CD18 and CD11c/CD18 do not compensate these metabolic alterations.

In summary, these findings suggested an important role of the functional β2-integrin CD11b/CD18 to coordinate the cell attachment and a subsequent monocytic maturation program upon phorbol ester induction. Thus, a block in CD11b/CD18-mediated adherence and regulatory imbalances can result in additional secondary effects after TPA stimulation, such as a derailed regulation of proliferation and a failed extracellular matrix restructuring by the lack of a sufficient production of matrix metalloproteinases. Moreover, reduced availability of distinct intracellular protease systems may reflect an insufficient adaptation to monocytic metabolism following induction of differentiation.

## Competing interests

The authors declare that they have no competing interests.

## Authors’ contributions

AO performed the telomerase assay and contributed to Figure 5 and Table 1. KM contributed to Figure 2. RH designed the study, contributed Figures 1b, 3, and 4 and drafted the manuscript. All authors have read and approved the final version of the manuscript.
